# Electroencephalographic sleep macrostructure and sleep spindles in early infancy

**DOI:** 10.1093/sleep/zsab262

**Published:** 2021-11-10

**Authors:** Soraia Ventura, Sean R Mathieson, John M O’Toole, Vicki Livingstone, Mary-Anne Ryan, Geraldine B Boylan

**Affiliations:** 1 Department of Paediatrics & Child Health, University College Cork, Cork, Ireland; 2 INFANT Research Centre, University College Cork, Cork, Ireland

**Keywords:** neurodevelopment, sleep, spindles, macrostructure, infant, EEG, REM, NREM, nap

## Abstract

**Study Objectives:**

Sleep features in infancy are potential biomarkers for brain maturation but poorly characterized. We describe normative values for sleep macrostructure and sleep spindles at 4–5 months of age.

**Methods:**

Healthy term infants were recruited at birth and had daytime sleep electroencephalograms (EEGs) at 4–5 months. Sleep staging was performed and five features were analyzed. Sleep spindles were annotated and seven quantitative features were extracted. Features were analyzed across sex, recording time (am/pm), infant age, and from first to second sleep cycles.

**Results:**

We analyzed sleep recordings from 91 infants, 41% females. Median (interquartile range [IQR]) macrostructure results: sleep duration 49.0 (37.8–72.0) min (*n* = 77); first sleep cycle duration 42.8 (37.0–51.4) min; rapid eye movement (REM) percentage 17.4 (9.5–27.7)% (*n* = 68); latency to REM 36.0 (30.5–41.1) min (*n* = 66). First cycle median (IQR) values for spindle features: number 241.0 (193.0–286.5), density 6.6 (5.7–8.0) spindles/min (*n* = 77); mean frequency 13.0 (12.8–13.3) Hz, mean duration 2.9 (2.6–3.6) s, spectral power 7.8 (4.7–11.4) µV^2^, brain symmetry index 0.20 (0.16–0.29), synchrony 59.5 (53.2–63.8)% (*n* = 91). In males, spindle spectral power (µV^2^) was 24.5% lower (*p* = .032) and brain symmetry index 24.2% higher than females (*p* = .011) when controlling for gestational and postnatal age and timing of the nap. We found no other significant associations between studied sleep features and sex, recording time (am/pm), or age. Spectral power decreased (*p* < .001) on the second cycle.

**Conclusion:**

This normative data may be useful for comparison with future studies of sleep dysfunction and atypical neurodevelopment in infancy.

**Clinical Trial Registration:** BABY SMART (Study of Massage Therapy, Sleep And neurodevelopMenT) (BabySMART)

URL: https://clinicaltrials.gov/ct2/show/results/NCT03381027?view=results.

ClinicalTrials.gov Identifier: NCT03381027

**Statement of Significance**

Sleep is fundamental at any stage of life but has particular importance during the first year. Sleep quality influences the architecture of neural connections and circuits, which are essential for memory consolidation and learning. During infancy, there is an unparalleled dynamic evolution of sleep that accompanies the progress of brain maturation. This study describes normative data for sleep macrostructure and sleep spindle features at 4 to 5 months and compares those parameters across sex, time of the day, first and second sleep cycle, and possible trends across gestational, postmenstrual, and postnatal ages, providing a benchmark for comparison with future studies of atypical neurodevelopment in infancy.

## Introduction

Some of the most important sleep waveforms recorded on the electroencephalogram (EEG) are sleep spindles. These transients, defined by the American Academy of Sleep Medicine (AASM) as runs of 11–16 Hz oscillatory activity occurring over central locations during N2 and N3 stages of sleep [1], arise from the thalamus [2] during a state of relative hyperpolarization [3]. Spindle properties are dependent on intra-thalamic interactions, particularly between the reticular nucleus and thalamocortical neurons, and amongst thalamic and cortical structures [4–7]. In humans, the first spindles can be detected on surface EEG as early as 6 to 9 weeks postnatal age (PNA) [8].

Since early analysis by Gibbs and Gibbs [9], it has been extensively demonstrated that there are two spindle frequencies: slow spindles, prevalent over the frontal areas, and fast sleep spindles, prevalent over the central/centroparietal areas, in children [10–12] and adults [13–17]. The two types of spindles correspond to different underlying dynamics [13, 17, 18], cortical and subcortical activations [19], and may reflect specific processes of learning and cognitive ability [13, 16, 18, 20–22]. Different studies have used different cut-off frequency points for the two types of sleep spindles, and this inconsistency may be related to interindividual differences [23].

Sleep spindle features are partially dependent on genetic factors [24], including sex [23, 25]. However, several environmental factors can influence sleep spindle parameters, including circadian rhythm and sleep pressure [23, 25–29]; activities where learning might be involved [30–35]; cognitive abilities; and pathological conditions such as Alzheimer’s [36], Parkinson’s [37], attention deficit hyperactivity disorder [38], autism spectrum disorder [39], schizophrenia [40, 41], and some cases of cerebral palsy [42]. A possible explanation linking some manifestations of neurological diseases to alterations in spindle parameters may reside in the fact that several “at-risk” genes for these pathologies are highly expressed in the thalamic reticular nucleus [43], which is integral to spindle generation.

With aging, there is an early shift of the maximal spindle-related power toward anterior locations [44], and evolution of several individual sleep spindle parameters such as density, frequency, duration, and amplitude [12, 25, 45]. The most marked changes occur during infancy but continue to a lesser extent until adulthood, accompanying major brain maturation processes, further to this, sleep spindles might present properties that actively contribute to some synaptic development [46] (for review see Ref. [47]).

In the pediatric population, slower and fast sleep spindles appear to play different roles in the varying factors that compose intelligence [10, 48–50]. However, no association was observed in a cohort from 12 to 30 months in a series of tests that included socialization, hand-and-eye coordination, and other motor functions [51]. Similar to the adult population [52], there has been no evidence that sleep spindles in children are related to verbal cognitive ability with the exception of narrative memory [48, 49]. Also, like adult studies, there seem to be sex differences regarding these associations in populations as young as 4 to 8 years old [10]. It has also been demonstrated that there are reciprocal interactions between sleep spindle characteristics such as number [30, 31, 34], density [32, 35, 53], spectral power [30, 33], amplitude [53], duration [53] and relationship with slow waves [18], and the ability to learning different tasks.

At term, the proportion of time spent in active sleep is at its highest [54] and sleep tends to initiate with this sleep stage. The shift to quiet sleep onset occurs after the 44th week of postmenstrual age (PMA) [55, 56]. During infancy, latency to rapid eye movement stage (REM) increases and becomes more dependent on duration of the previous wakefulness period [57], while the proportion of active to quiet sleep and, subsequently, REM:non-rapid eye movement (NREM) sleep continues to decrease [54, 58]. In infancy, REM:NREM proportions from daytime naps maturate earlier relative to nocturnal proportions [59]. From 4 months of age, NREM sleep can be further classified into stage 1 (N1), stage 2, (N2), and stage 3 (N3), as the stages become increasingly differentiated [60].

Sleep is theorized to endorse homeostatic balance at the cellular level [61] and to prevent neuroinflammation [62–64]. Sleep reshapes the maturing brain [65, 66]; in a period of life with less efficient neural networks, sleep in thought to contribute to a general energy saving by reducing the number of redundant synapses, and enhancing strategic synapses, thus promoting learning and memory consolidation [63]. It allows somatosensory and motor development [67, 68], prolongs stimuli recognition in infants [69–71], and contributes to mood regulation [72]. Poor quality of sleep during development is associated with higher risk for the development of psychopathological symptoms [73] and lower cognitive outcomes later in life [62].

The process of neurodevelopment during the first months of life is dynamic with changes in sleep macrostructure and sleep spindles reflecting some of these alterations. These features may provide early biomarkers for development. Sleep spindles, in particular, show a unique individual *fingerprint* [15] and are affected by different neurologic pathologies. The normative values for the 4 to 5-month age group may offer a benchmark with which to assess a potentially abnormal developmental trajectory. In this study, we analyze the macrostructure of sleep, as well as sleep spindles of ex-term infants at 4 to 5 months of age, possible influences of gender, period of the day the nap occurs, and differences from first to second sleep cycle. The sleep parameter trends are also analyzed across gestational age (GA), PMA, and PNA.

## Methods

This study was approved by the Clinical Research Ethics Committee of the Cork Teaching Hospitals. The guardians of the participants were approached for consent in the postnatal ward at Cork University Maternity Hospital between 2017 and 2018. The criteria for inclusion were being healthy, singleton, and born after 37 weeks of GA.

Infants were enrolled into the BabySMART study, a randomized controlled study investigating the impact of an intervention (infant massage) on sleep and neurodevelopment in early infancy. EEG analysis was performed blinded to group allocation. Only the data from the nonintervention group was used in this analysis. Infants participated in a sleep EEG recording at approximately 4–5 months of age. Appointments occurred during the day, either during the morning or the afternoon, generally between 9.30 and 12.00 am, and 1.30 and 4.00 pm respectively. The soundproof room was at a comfortable temperature according to parents’ judgment and lights dimmed; guardians could opt to let the infant sleep on their lap, the buggy, or in a cot. During EEG electrode placement, feeding was encouraged to distract the infants from the procedure and soothe them.

### EEG recording

Video-EEGs were recorded using a Lifelines (Lifelines Neuro, UK) EEG acquisition system. Electrode placement followed the international 10–20 system and included FP2, FP1, F8, F7, F4, F3, Fz, A2, A1, T4, T3, C4, C3, Cz, T8, T7, P4, P3, Pz, O2, O1, reference, and ground. Disposable cup EEG electrodes were used and impedances were maintained below 10 kΩ. Additional polygraphy, including electrocardiogram, electrooculogram, submental electromyogram, and respiration, was recorded. The EEG was sampled at 500 Hz. The EEG was recorded for the sleep duration and finished after the baby fully woke and was no longer able to return to sleep. A clinical physiologist remained in the room during the recording to promptly address any technical issues that might arise and to flag any incidental EEG findings.

### EEG analysis

The post-acquisition EEG analysis of sleep macrostructure and sleep spindles was performed by an experienced clinical physiologist (S.V.). EEG was reviewed using standard high- and low-pass filters of 0.5 and 70 Hz respectively.

Sleep staging was performed according to the AASM guidelines version 2.4, in 30-second epochs and annotated using sleep staging software (Nicolet, Natus). Sleep spindles present over F4-C4 and F3-C3 EEG channels were visually identified post-acquisition on Stratus EEG (Kvikna, Iceland) software and were marked on the EEG with duration annotations, populating an events list that could be exported as a text file for further analysis. The spindle parameters analyzed were the number of spindles, density (number of sleep spindles per minute of NREM), duration (s), median sleep spindle spectral power (µV^2^), mean spindle frequency (Hz), brain symmetry index [74] (a measure of divergence of inter-hemispheric power), and synchrony. This data was segregated by sleep cycles. Spectral power and mean frequency were calculated from power spectral density (PSD) estimates of each spindle. These measures used a periodogram PSD. Brain symmetry index was calculated over a 30 s epoch with 75% overlap; non-spindle activity in this epoch was ignored. This measure used a Welch PSD with a 0.5 s Hamming window with 75% overlap. All PSD estimates were limited to the range 9 to 16 Hz and spindles of <0.5 s in duration were ignored. The median values of number of spindles, duration, median spindle spectral power, and mean spindle frequency were calculated as a summary measure per infant. Synchrony, a measure of coincidental contralateral sleep spindles, was calculated as the percentage overlap between spindles in the left and right hemispheres. Duration of spindles was obtained from the sleep spindles individual annotations and density was calculated based on the number of sleep spindles and the duration of NREM sleep during each sleep cycle. Mean frequency and synchrony were calculated using Matlab (The Mathworks Inc, Natick, MA, version 9.8.0 R2020a); brain symmetry index and spectral power of the spindles were calculated using NEURAL software (a neonatal EEG feature set in Matlab, version 0.4.3) [75].

Griffiths III is a neurodevelopmental assessment tool, designed to be administered to children from birth to 5 years and 11 months [76]. Infants with an overall Griffiths developmental quotient (GDQ) of 85 or lower at 18 months were considered to have developmental delay and were excluded from this study. Infants were also excluded if their GDQ score was not available at 18 months, a sleep recording was not obtained or EEG abnormalities were found. Further exclusions were applied depending on the amount of sleep data recorded. As there were some infants that fell asleep before the recording initiated, those infants were excluded for calculations of spindle number and density due to the potential for missing data. Only infants with a complete first sleep cycle were considered for the macrostructure analyses. Here, complete sleep cycle meant that infants only fell asleep after the onset of the polysomnography and had both NREM and REM sleep, with a requirement for N2 and/or N3 during NREM state being present. We noted exceptional cases where REM sleep occurred before meeting criteria for N2; to avoid joining the norm with the exception on the measures of latency to REM, these infants were excluded from these specific variable analyses. For comparison between first and second cycle, the previous rules still applied on the micro- and macrostructure; additionally, for microstructure analysis, we included infants who had NREM recorded for both sleep cycles, and for macrostructure, infants who had at least two complete sleep cycles.

### Statistical analysis

Statistical analyses were performed using IBM SPSS Statistics (version 26.0, IBM Corp., Armonk, NY). Continuous variables were described using mean and standard deviation (SD) or median and interquartile range (IQR) and categorical variables were described using frequency and percentage. Relationships between continuous variables were initially investigated using Pearson’s correlation coefficient.

Univariable and multivariable linear regression was used to investigate factors associated with sleep features. The factors investigated were age (GA and PNA), sex, and period of the day. Some sleep features were log-transformed prior to the regression analyses: spindle spectral power and brain symmetry index and macrostructural metrics of time in REM, first cycle duration, latency to sleep, that is, time from the period of lights off at the start of EEG to onset of sleep, and to REM variables. The regression coefficients (*B*) and their corresponding 95% CIs were back-transformed and presented in the original units.

Differences in sleep parameters between the first and second sleep cycle were investigated using the Wilcoxon signed-rank test. All tests were two-sided and a *p*-value < .05 was considered statistically significant. Sleep spindles annotations analyzed in this study were compared with annotations of a second-rater using the Kappa statistic.

## Results

**Figure 1.**
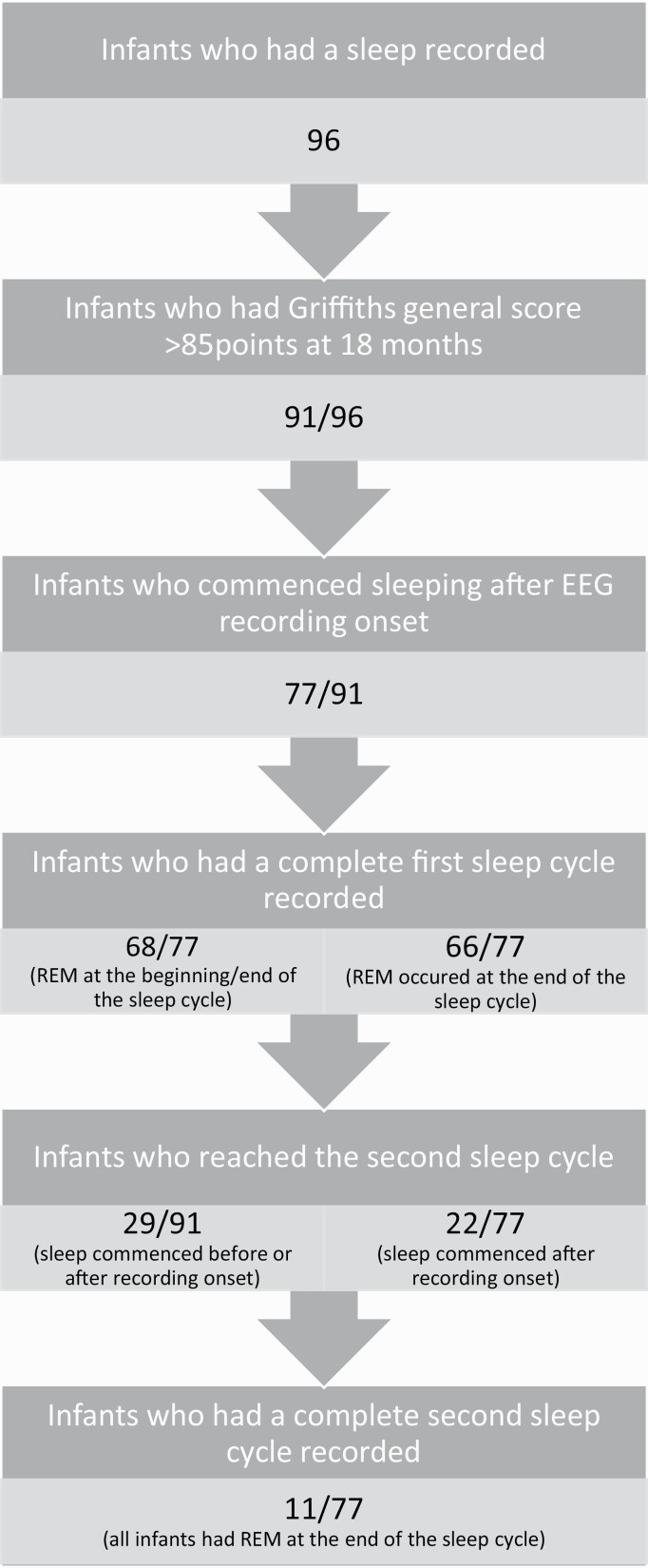
Flow diagram detailing numbers of participants in analysis.

Ninety-eight infants had an EEG recording at 4 to 5 months of age. Of those, two were excluded, as one did not fall asleep and the other had an abnormal EEG. We further excluded four infants who did not have a Griffith’s-III neurodevelopmental assessment at 18 months and one infant whose GDQ score was equal or lower than 85 (Figure 1). Table 1 describes the demographic information of the 91 infants included in this study.

**Table 1.** Demographics

Characteristics	Mean (SD)* (*n* = 91)
Sex—female, *n* (%)	37 (40.7)
Weight at birth (kg)	3.57 (0.46)
Gestational age (weeks)	39.8 (1.2)
PNA at the appointment (weeks)	19.5 (1.3)
PMA at the appointment (weeks)	59.3 (1.8)
Timing of the nap—afternoon, *n* (%)	49 (53.8)
Mother’s ethnicity, *n* (%)	
White Irish	82 (90.1)
Non-Irish White	6 (6.6)
Asian	2 (2.2)
Latin-American	1 (1.1)
Father’s ethnicity, *n* (%)	
White Irish	82 (90.1)
Non-Irish White	6 (6.6)
Asian	2 (2.2)
Arabic	1 (1.1)

Gestational age is referred as the time from mothers’ last menstrual cycle to delivery, PNA as age at the occasion of the EEG recording counted from birth and postmenstrual age as the sum of both.^94^

*Unless otherwise stated.

PNA, postnatal age; PMA, postmenstrual age.

### Analysis of diurnal sleep macrostructure

A median (IQR) total recording time of 65.0 (49.5–87.5) min was obtained for 91 infants in the study; 42 recordings (46.2%) occurred during the morning time. In total, 14 infants fell asleep during electrode application. The total nap duration for those who fell asleep after the recording started had a median (IQR) of 49.0 (37.8–72.0) min (*n* = 77). Of those 77 infants, 68 had a complete first sleep cycle recorded. Twenty-nine infants out of the total 91 reached the second sleep cycle. When including infants that fell asleep after the recording started (*n* = 77), only 22/77 (28.6%) reached at least the second sleep cycle; 5/77 (7.8%) achieved and 3/77 (3.9%) completed the third cycle. 1/77 infant reached the 4th sleep cycle (Figure 1). Two infants out of 77 (2.6%) reached REM stage before meeting the criteria for N2.

**Figure 2.**
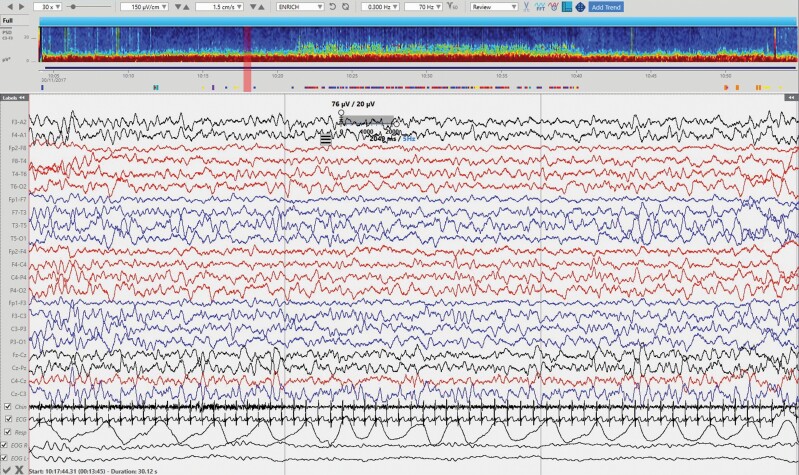
N1 sleep stage with its characteristic relative low amplitude and mixed frequencies and slow eye movement (intrusion of frontal activity is present on the EOG channels). Incipient vertex sharp waves are present in the central regions. Gridlines represent 10 s-intervals. Power spectrum density (PSD) window on top of the EEG represent spectral power of frequencies from 0.5 to 30 Hz (*y* axis) across the approximately 1 h of recording (*x* axis). Red colors represent the highest spectral power for a particular frequency and time on the spectrum; colors close to dark blue represent the lower spectral power.

**Figure 3.**
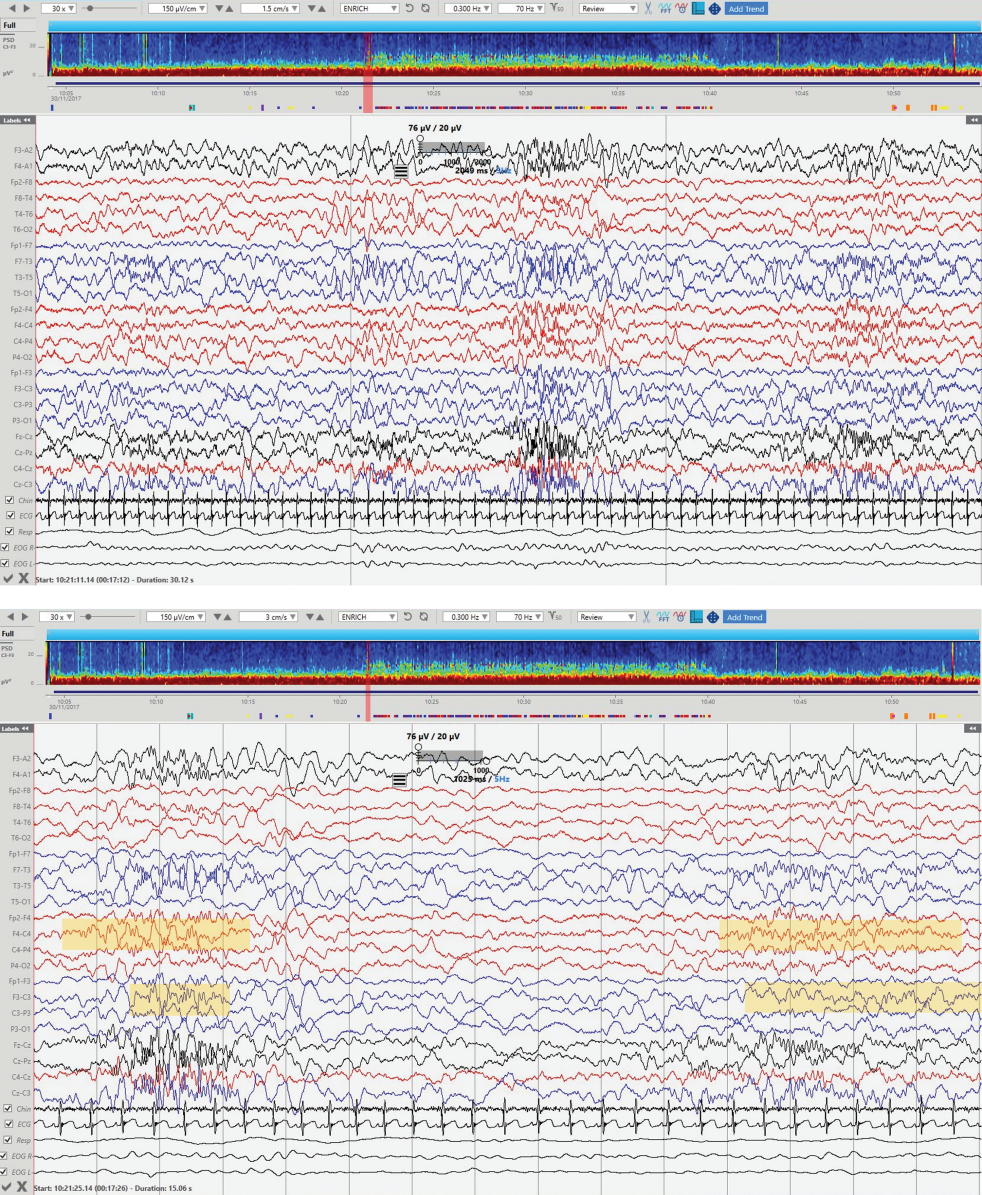
N2 sleep stage. Sleep spindles marked the onset of this epoch. Top EEG: timebase = 1.5 cm/s, gridlines correspond to 10 s intervals. Bottom EEG: detail of the previous page to evidence spindles, timebase = 3cm/s, gridlines correspond to 1 s intervals. Right and left fronto-central sleep spindles in yellow.

**Figure 4.**
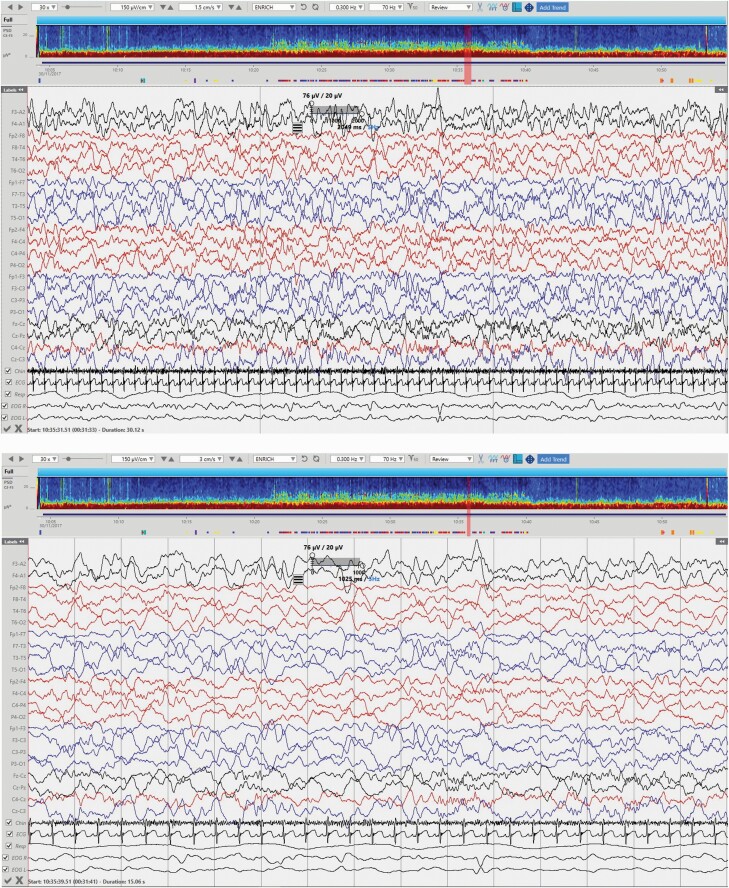
N3 sleep stage. High amplitude slow waves are a hallmark of this stage (intrusions of frontal high amplitude activity present on the EOG channels). Top EEG: timebase =1.5cm/s, gridlines denote 10 s intervals. Bottom EEG: detail of the previous page, timebase = 3cm/s, gridlines denote 1 s intervals.

**Figure 5.**
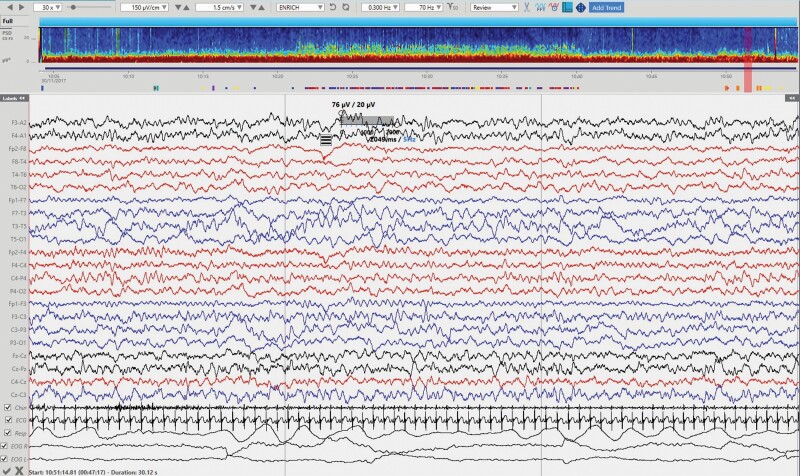
REM sleep stage with relatively low amplitude activities, accompanying conjugate rapid eye movements, irregular respiration rate and lower chin EMG.

Table 2 represents the duration of sleep stages during the first complete cycle and latencies to sleep and REM (*n* = 68). Figures 2–5 illustrate the typical sleep stages from N1 to REM of a participant.

**Table 2.** Duration of sleep stages on the first cycle

Sleep stages	Median (IQR) (*n* = 68)	
	Time (minutes)	Time (% from cycle duration)
Latency to sleep	8.5 (4.0–14.0)	—
Cycle duration	42.8 (37.0–51.4)	—
NREM	35.5 (30.1–40.5)	82.6 (72.3–90.5)
N1	8.0 (4.5–13.4)	18.8 (13.3–28.0)
N2	4.5 (2.5–6.5)	10.9 (6.0–16.5)
N3	20.3 (16.5–25.4)	46.0 (38.3–59.7)
REM	6.5 (3.6–11.5)	17.4 (9.5–27.7)
Latency to REM*	36.0 (30.5–41.1)	—

*n = 66, infants who meet criteria for staging REM before N2 were excluded.

Of the 96 infants with a clinically normal EEG and with sleep recorded, 5 were excluded from the study due to lacking or having a low score on Griffiths and, 15 were excluded from macrostructure analysis due to falling asleep before the recording onset. As an indicator of sample size calculation for future studies, ignoring the mentioned exclusions, 72/96 infants (75.0%) reached a complete sleep cycle (N1 may not have been recorded). Two further infants had REM before reaching N2 and N3 out of the 81 whose sleep onset was recorded (2.5%).

### Analysis of diurnal sleep spindles

**Figure 6.**
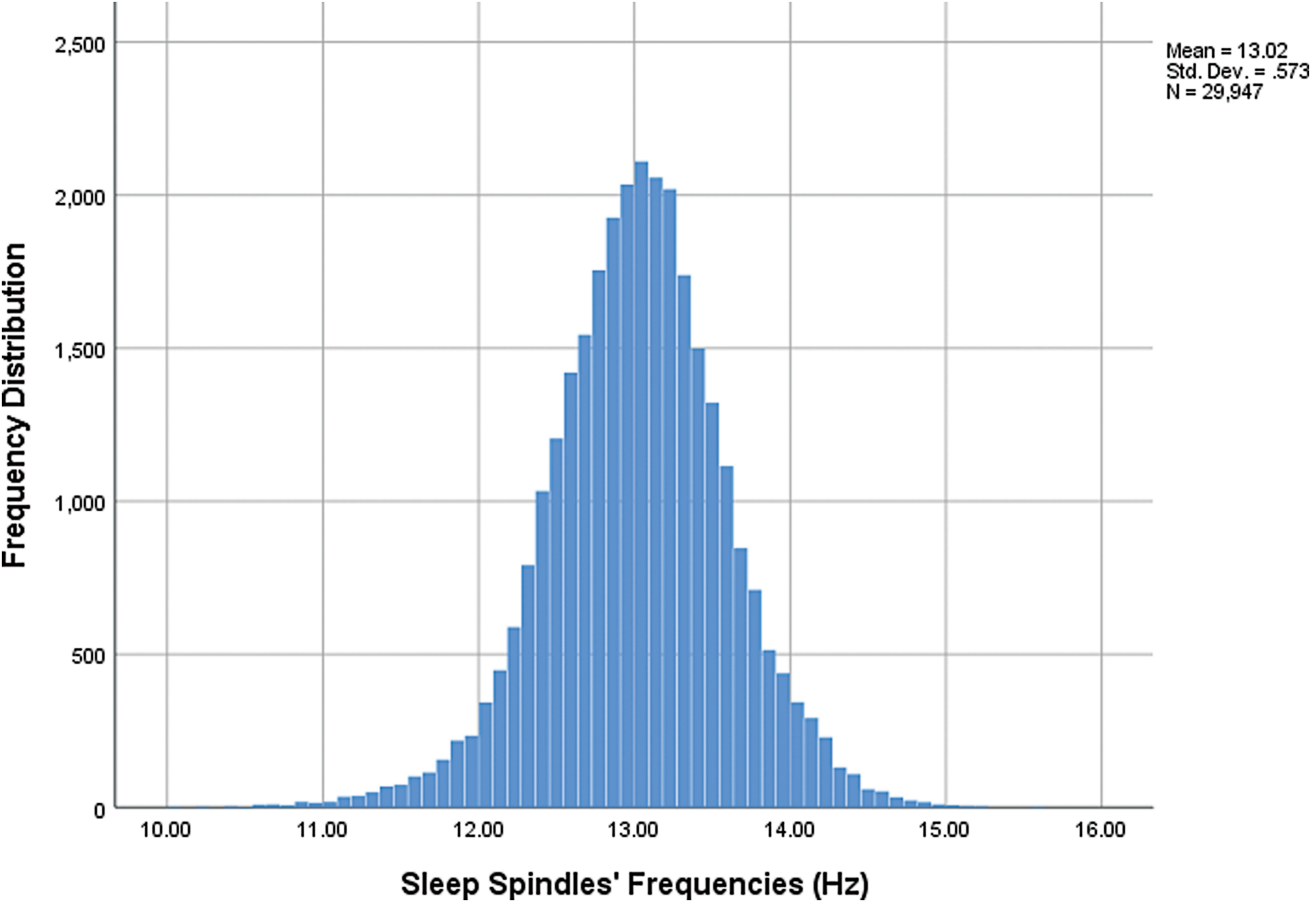
Distribution of sleep spindle frequencies (Hz) from all sleep cycles recorded.

To calculate interobserver agreement for detection of sleep spindles, the first sleep cycles of 65/96 infants were annotated by two raters and compared. Kappa scores for spindle identification were 0.82 and 0.80 for right and left fronto-central spindles respectively, which confirm that sleep spindles are discrete and readily identifiable waveforms. 50.3% of the sleep spindles were right-sided. From the 91 participants, we recorded a total of 29 947 sleep spindles. For the first cycle, 21 867 spindles were recorded. Table 3 provides the characterization of the sleep spindles during the first sleep cycle across infants. The distribution of sleep spindle frequencies (Hz) is shown in Figure 6.

**Table 3.** Analysis of sleep spindle features during the first sleep cycle

Sleep spindle parameters	Median (IQR) (*n* = 91)
Frequency (Hz)	13.0 (12.8–13.3)
Duration (s)	2.9 (2.6–3.6)
Spectral Power (µV^2^)	7.8 (4.7–11.4)
Brain symmetry index	0.20 (0.16–0.29)
Synchrony (%)	59.5 (53.2–63.8)
Number*	241.0 (193.0–286.5)
Density* (spindles/min)	6.6 (5.7–8.0)

*Infants who fell asleep before EEG started were excluded (*n* = 77).

### Sleep parameter regression analysis

Results from the univariable and multivariable linear regression analyses between sleep spindle parameters at 4 to 5 months during the first sleep cycle and GA, PNA, sex, and timing of the nap are presented in Table 4. In the univariable analysis, GA was negatively correlated with synchrony [*B*(95% CI): −1.307(−2.583 to –0.031), *p* = .045] but this relationship was no longer statistically significant in multivariable analysis [*B*(95% CI): −1.216(−2.547 to 0.115), *p* = .073]. Likewise, PMA and spindle duration were negatively correlated [B(95% CI): −0.086 (−0.158 to −0.015), *p *= .018] in the univariable analysis, losing statistical significance when controling for sex and timing of the nap (results not included). Sleep spindle spectral power and brain symmetry index differed by sex in the univariable analyses with significantly higher power and lower brain symmetry index in females (*p* = .022 and *p* = .010, respectively). These differences remained statistically significant in the multivariable analyses (*p* = .032 and *p* = .011, respectively). Comparisons of sleep parameters by timing of the nap (morning or afternoon) demonstrated lower spindle duration during afternoons in the unadjusted analysis (*p* = .034) but the difference was not significant in the adjusted analysis (*p* = .115). No significant relationships between the factors and the macrostructure outcomes were found in the univariable or multivariable analyses (Table 5).

**Table 4.** Univariable and multivariable linear regression analysis of the sleep spindle features on GA, PMA, PNA, sex, and timing of the nap

			Univariable analysis (*n* = 91)				Multivariable analysis (*n* = 91)		
Dependent Variable	Independent Variable		*r*/median (IQR)	*B*	95% CI	*P* †	*B*	95% CI	*P* ‡
Frequency (Hz)	GA		0.024	0.007	(–0.053 to 0.066)	.825	–0.004	(–0.066 to 0.058)	.906
	PMA		0.066	0.013	(–0.028 to 0.054)	.534			
	PNA		0.068	0.018	(–0.038 to 0.074)	.520	0.010	(–0.048 to 0.068)	.723
	Sex	Female (ref)	13.1 (12.8 to 13.3)						
		Male	13.0 (12.8 to 13.2)	–0.051	(–0.197 to 0.096)	.494	–0.041	(–0.191 to 0.110)	.593
	Timing of the nap	Morning (ref)	13.0 (12.7 to 13.2)						
		Afternoon	13.0 (12.9 to 13.3)	0.101	(–0.042 to 0.244)	.165	0.098	(–0.053 to 0.249)	.202
Duration (min)	GA		–0.169	–0.086	(–0.192 to 0.020)	.109	–0.064	(–0.171 to 0.043)	.240
	PMA		–0.246*	–0.086	(–0.158 to –0.015)	.018*			
	PNA		–0.179	–0.086	(–0.185 to 0.014)	.090	–0.068	(–0.168 to 0.033)	.185
	Sex	Female (ref)	2.7 (2.6 to 3.5)						
		Male	3.1 (2.6 to 3.6)	0.158	(–0.104 to 0.421)	.233	0.109	(–0.152 to 0.370)	.409
	Timing of the nap	Morning (ref)	3.2 (2.7 to 3.7)						
		Afternoon	2.8 (2.6 to 3.4)	–0.276	(–0.530 to –0.022)	.034*	–0.210	(–0.473 to 0.052)	.115
Spectral Power (µV^2^)§	GA		0.168	1.088	(0.980 to 1.206)	.112	1.079	(0.970 to 1.198)	.158
	PMA		0.167	1.059	(0.986 to 1.137)	.113			
	PNA		0.072	1.034	(0.937 to 1.141)	.500	1.010	(0.915 to 1.115)	.839
	Sex	Female (ref)	8.9 (5.9–12.2)						
		Male	6.4 (4.3–10.3)	0.746	(0.581 to 0.958)	.022*	0.755	(0.584 to 0.976)	.032*
	Timing of the nap	Morning (ref)	7.2 (4.2–10.3)						
		Afternoon	8.2 (5.0–11.6)	1.119	(0.868 to 1.442)	.380	1.052	(0.813 to 1.361)	.697
BSI§	GA		0.090	1.029	(0.962 to 1.102)	.396	1.021	(0.954 to 1.093)	.545
	PMA		0.017	1.004	(0.958 to 1.051)	.875			
	PNA		–0.062	0.981	(0.920 to 1.046)	.561	0.993	(0.931 to 1.058)	.822
	Sex	Female (ref)	0.18 (0.16–0.24)						
		Male	0.24 (0.17–0.32)	1.239	(1.054 to 1.456)	.010*	1.242	(1.052 to 1.465)	.011*
	Timing of the nap	Morning (ref)	0.19 (0.16–0.27)						
		Afternoon	0.22 (0.17–0.30)	1.108	(0.941 to 1.305)	.216	1.108	(0.939 to 1.309)	.222
Synchrony	GA		−0.211*	−1.307*	(–2.583 to –0.031)	.045*	–1.216	(–2.547 to 0.115)	.073
	PMA		−0.144	−0.613	(–1.500 to 0.273)	.173			
	PNA		0.001	0.004	(–1.223 to 1.231)	.994	–0.067	(–1.313 to 1.178)	.915
	Sex	Female (ref)	60.4 (53.4 to 64.6)						
		Male	59.1 (53.0 to 63.6)	−1.386	(–4.585 to 1.813)	.392	–1.547	(–4.785 to 1.690)	.345
	Timing of the nap	Morning (ref)	61.4 (54.6 to 65.0)						
		Afternoon	58.2 (52.2 to 63.7)	−1.724	(–4.868 to 1.420)	.279	–1.047	(–4.301 to 2.206)	.524
Number‖	GA		0.095	5.787	(–8.203 to 19.777)	.413	3.410	(–11.072 to 17.892)	.640
	PMA		–0.050	−2.180	(–12.103 to 7.743)	.663			
	PNA		–0.157	−9.078	(–22.204 to 4.048)	.172	–9.648	(–23.303 to 4.007)	.163
	Sex	Female (ref)	237.5 (179.0 to 305.8)						
		Male	245.0 (208.0 to 285.0)	–0.200	(–34.134 to 33.734)	.991	–4.750	(–39.596 to 30.095)	.787
	Timing of the nap	Morning (ref)	230.5 (192.8 to 287.3)						
		Afternoon	253.0 (200.0 to 287.0)	18.152	(–14.911 to 51.214)	.278	17.448	(–16.737 to 51.632)	.312
Density (spindles/min)‖	GA		0.015	0.021	(–0.307 to 0.348)	.900	0.006	(–0.340 to 0.351)	.974
	PMA		–0.009	–0.009	(–0.240 to 0.223)	.940			
	PNA		–0.025	–0.034	(–0.344 to 0.276)	.826	–0.048	(–0.373 to 0.278)	.771
	Sex	Female (ref)	6.9 (5.3 to 8.8)						
		Male	6.6 (5.8 to 7.9)	–0.117	(–0.908 to 0.674)	.769	–0.142	(–0.972 to 0.689)	.735
	Timing of the nap	Morning (ref)	6.7 (5.3 to 8.0)						
		Afternoon	6.6 (5.8 to 8.1)	0.081	(–0.696 to 0.857)	.837	0.082	(–0.733 to 0.897)	.842

Pearson correlation coefficient (*r*); reference variable (ref); females: *n* = 37; males: *n* = 54; morning: *n* = 42; afternoon: *n* = 49, unless stated otherwise.

*P < .05.

^†^From simple regression analysis.

^‡^From multiple regression analysis..

^§^Log-transformed prior to analysis and then back-transformed.

^‖^Infants who fell asleep before EEG were excluded (*n* total= 77; females: *n* = 30; males: *n* = 47; morning: *n* = 34; afternoon: *n* = 43).

**Table 5.** Univariable and multivariable linear regression analysis of the sleep macrostructure features on GA, PMA, PNA, sex, and timing of the nap

			Univariable analysis (*n* = 68)				Multivariable analysis (*n* = 68)		
			*r*/median (IQR)	*B*	95% CI	*P* †	*B*	95% CI	*P* ‡
Time in NREM (min)§	GA		0.029	1.006	(0.955 to 1.060)	.814	1.003	(0.951 to 1.059)	.905
	PMA		–0.095	0.986	(0.951 to 1.022)	.443			
	PNA		–0.166	0.965	(0.917 to 1.016)	.177	0.966	(0.915 to 1.019)	.197
	Sex	Female (ref)	36.5 (26.0–40.5)						
		Male	34.5 (30.5–42.8)	1.041	(0.916 to 1.184)	.533	1.025	(0.899 to 1.170)	.707
	Timing of the nap	Morning (ref)	34.0 (29.5–39.5)						
		Afternoon	36.0 (30.2–43.0)	1.051	(0.928 to 1.192)	.429	1.055	(0.926 to 1.201)	.414
Time in REM (min)	GA		–0.030	–0.157	(–1.436 to 1.122)	.807	–0.181	(–1.522 to 1.161)	.789
	PMA		0.055	0.199	(–0.695 to 1.094)	.658			
	PNA		0.109	0.571	(–0.710 to 1.852)	.377	0.531	(–0.804 to 1.866)	.430
	Sex	Female (ref)	7.5 (4.4 to 13.6)						
		Male	6.2 (2.9 to 11.5)	–0.870	(–4.039 to 2.299)	.585	–0.674	(–3.976 to 2.628)	.685
	Timing of the nap	Morning (ref)	7.5 (4.5 to 11.5)						
		Afternoon	6.0 (3.5 to 11.5)	–0.218	(–3.317 to 2.881)	.889	–0.198	(–3.436 to 3.041)	.903
%REM	GA		–0.014	–0.118	(–2.196 to 1.960)	.910	–0.053	(–2.193 to 2.087)	.961
	PMA		0.104	0.617	(–0.830 to 2.064)	.397			
	PNA		0.164	1.397	(–0.667 to 3.461)	.181	1.284	(–0.847 to 3.414)	.233
	Sex	Female (ref)	17.7 (10.7 to 29.1)						
		Male	17.2 (7.5 to 25.8)	–2.899	(–8.008 to 2.209)	.261	–2.315	(–7.583 to 2.954)	.383
	Timing of the nap	Morning (ref)	18.0 (10.7 to 28.0)						
		Afternoon	17.1 (7.9 to 26.9)	–1.970	(–6.979 to 3.039)	.435	–2.120	(–7.288 to 3.047)	.415
Cycle duration (min)§	GA		0.021	1.004	(0.953 to 1.058)	.866	1.002	(0.949 to 1.059)	.937
	PMA		–0.042	0.994	(0.958 to 1.031)	.735			
	PNA		–0.081	0.983	(0.932 to 1.036)	.511	0.982	(0.930 to 1.037)	.506
	Sex	Female (ref)	44.0 (35.1–52.4)						
		Male	41.8 (37.4–51.1)	1.007	(0.884 to 1.146)	.920	0.999	(0.872 to 1.143)	.983
	Timing of the nap	Morning (ref)	43.5 (36.5–51.0)						
		Afternoon	42.5 (37.5–51.8)	1.028	(0.906 to 1.166)	.663	1.030	(0.902 to 1.176)	.660
Latency to Sleep (min)§	GA		0.071	1.062	(0.863 to 1.306)	.566	1.016	(0.821 to 1.258)	.880
	PMA		0.094	1.057	(0.915 to 1.221)	.448			
	PNA		0.063	1.055	(0.857 to 1.300)	.608	1.028	(0.831 to 1.272)	.795
	Sex	Female (ref)	8.8 (3.5–23.9)						
		Male	7.5 (4.7–13.6)	0.800	(0.479 to 1.334)	.387	0.810	(0.479 to 1.372)	.428
	Timing of the nap	Morning (ref)	7.0 (4.0–12.0)						
		Afternoon	9.0 (3.5–23.2)	1.470	(0.897 to 2.406)	.124	1.453	(0.867 to 2.434)	.153
Latency to REM (min)§,‖	GA		0.133	1.032	(0.973 to 1.095)	.289	1.026	(0.965 to 1.092)	.404
	PMA		–0.010	0.998	(0.958 to 1.041)	.936			
	PNA		–0.147	0.966	(0.910 to 1.024)	.239	0.963	(0.907 to 1.024)	.222
	Sex	Female (ref)	36.8 (28.9–42.0)						
		Male	35.0 (30.6–40.8)	1.010	(0.871 to 1.170)	.898	1.000	(0.860 to 1.161)	.996
	Timing of the nap	Morning (ref)	35.0 (29.0–40.0)						
		Afternoon	36.5 (30.5–43.5)	1.069	(0.927 to 1.234)	.353	1.060	(0.913 to 1.231)	.436

Pearson correlation coefficient (*r*); reference variable (ref); females: *n* = 26; males: *n* = 42; morning: *n* = 31; afternoon: *n* = 37.

^†^From simple regression analysis.

^‡^From multiple regression analysis.

^§^Log-transformed prior to analysis and then back-transformed.

^‖^Infants who did not have a complete sleep cycle recorded or meet criteria for staging REM before N2 were excluded (*n* total = 66; females: *n* = 26; males: *n* = 40, morning: *n* = 31; afternoon: *n* = 35).

To study sleep macrostructure across the first two cycles, further exclusion was made to only include infants who had two complete sleep cycles (*n* = 11). No statistically significant differences were observed in sleep macrostructure parameters between cycles (Table 6). Changes in sleep spindle parameters along with the reduction of sleep pressure from the first to second cycle were investigated using the data of 29 infants who reached a second sleep cycle. Sleep spindle spectral power decreased significantly on the second cycle with (*p* < .001, Table 6).

**Table 6.** Comparison of macrostructure and sleep spindle parameters' values from first to second sleep cycles

	Median (IQR)		*P* †
	First cycle	Second cycle	
Macrostructure analysis (*n* = 11)‡			
Time in NREM (min)	28 (20.0–36.0)	30 (27.5–37.0)	.285
Time in REM (min)	10.5 (4.5–14.5)	7.5 (3.5–12.5)	.533
%REM	29.7 (13.8–33.0)	16.7 (11.6–29.4)	.213
Cycle duration (min)	37 (29.0–43.0)	40.5 (34.5–45.0)	.929
Latency to REM (min)	28.5 (21.5–36)	31 (27.5–37.5)	.213
Sleep spindle analysis (*n* = 29)			
Frequency (Hz)	13 (12.8–13.2)	13 (12.8–13.3)	.689
Duration (s)	2.9 (2.6–3.6)	2.9 (2.7–3.4)	.496
Spectral power (µV^2^)	8.2 (4.9–14.5)	6.4 (4.4–12.2)	<.001*
Brain Symmetry Index	0.18 (0.16–0.26)	0.19 (0.16–0.26)	.627
Synchrony (%)	58.2 (52.6–61.6)	57.2 (52.6–61.6)	.358
Number§	227 (174.2–246.5)	237.5 (170.0–302.5)	.200
Density§ (spindles/min)	6.4 (5.4–7.6)	7.6 (6.1–9.2)	.082

^†^From Wilcoxon test for paired samples.

^‡^Infants who did not have two complete sleep cycles recorded were excluded.

^§^ *n* = 22 due to additional exclusion of infants who fell asleep during electrode placement.

*P* < .05 marked with an asterisk (*).

## Discussion

We have described sleep macrostructure and sleep spindle parameters in the largest cohort to date of infants from 4 to 5 months of age. Sleep spindle spectral power decreased from the first to the second sleep cycle. From multivariable analysis, we noted that two-spindle features differed by gender: spindle spectral power and symmetry. There were no significant correlations between spindle features and time of recording (am/pm), GA and chronological age. No association were observed between sleep macrostructure analysis and sex, time of recording (am/pm), GA, and chronological age. This data serves as baseline normative values, which can be used in future studies to compare with infants at risk of abnormal neurodevelopment.

### Normative values for sleep macrostructure and sleep spindles at 4 to 5 months and their maturational evolution

Around the 44th week of PMA, sleep onset starts shifting from REM to NREM sleep [55]. In this study, only two infants reached REM sleep before reaching N2. As reviewed by de Weerd *et al*. [77], this is in keeping with other studies that did note a complete REM onset of sleep in some infants of this age group. Schulz *et al*. hypothesized that increased latency to REM in infancy is dependent on the establishment of the circadian rhythms through means of body temperature circadian amplitude variation and reduction of sleep fragmentation [57].

In the present study, the median (IQR) sleep latency was 8.5 (4.0–14.0) min [mean (SD) of 12.7 (15.8) min]. This latency is shorter in comparison with studies based on parent perception [78, 79]. Our results for sleep latency are similar to other studies when compared using the mean instead of the median (we opted to use the median as sleep latency data has a skewed distribution). Considering the high SD of the latency to sleep in the discussed papers and of the present study, it is clear that there is a high variance in sleep latencies for this age group which may further explain the different results. Additionally, differences in sleep latency may be explained by our use of polysomnographic recordings which provide a more accurate and objective measure of sleep onset, with all our recordings carried out in a temperature-controlled low noise sleep lab. At the same time, feeding during electrode placement in our study may have had a soothing effect that increased the infants’ predisposition to fall asleep, even before turning off lights and suspension of other stimuli. In fact, 14 infants fell asleep during the lights-on period while electrodes were being placed.

To date, the majority of studies seeking to characterize the evolution of sleep spindle parameters during the first year of life have included only small cohorts of infants. Nevertheless, these pioneer studies allow insight into the trends and values of different sleep spindle parameters during this neurodevelopmentally rich phase of life. During the first 6 months, the increasingly dominant sleep spindle frequencies range from 12.0 to 14.0Hz [8]. This finding is in accordance with our study, in which the median sleep spindle frequency was 13.0Hz with a narrow IQR of 12.8 to 13.3Hz, suggesting it is well conserved across infants. In contrast, spindles of slower rhythms (<12 Hz) become significantly less common across the same period [8]. Particularly after the first year, and at least, until reaching the 4-year mark, slow spindles reverse this tendency and increase in density [12]. It is widely accepted that two individual types of sleep spindles develop during childhood; fast spindles over more centroparietal areas and slow spindles over more anterior areas, that have different dynamics [19, 80] and subserve different functions (see for example Refs. [33, 53]). In the present study, the fronto-central channels were chosen to manually annotate the sleep spindles due to their predominance in this area on EEG visual inspection. However, if two sleep spindle types with distinct frequencies were present in these EEGs, we would expect some level of intrusion of fast sleep spindles through central electrodes and to be able to detect these two distinct frequency peaks on these channels. However, the histogram of the distribution of mean frequencies of each spindle (Figure 6), does not show obvious evidence of a second peak. The infants’ young age may explain the absence of a clear double peak and thus, its presence may constitute a marker of neurodevelopment. This finding is consistent with previous observations which suggest that the two types of sleep spindles do not coexist before the first year of life [12, 51]. Nevertheless, it remains a possibility that the two potential peaks were concealed by intra-individual differences, driven by plotting N2 and N3 spindles simultaneously, and inter-individual differences in frequency [15, 80] and further investigation on this matter may be required.

In the first 4 years, sleep spindle density, is highest between 4 and 12 months [12]. Sleep spindle synchrony and asynchrony values fluctuate during the first year with the highest sleep spindle asynchrony in earlier infancy [8, 81]. Symmetry is expected to be lowest between 4 and 5 months of age [8]. We identified two previous studies addressing spindle synchrony and one on spindle symmetry in infants [8, 81]. However, in our study, we used different methods to calculate symmetry and synchrony to these studies and our results may not be directly comparable. In the former studies, spindles were considered asymmetrical if one of the spindles were twice the amplitude of the contralateral side and were considered asynchronous if two contralateral spindle mid-points were 2s apart. We used the brain symmetry index which has been used before in adults [82] and we consider it to be a more accurate metric of spindle symmetry than the binary system used previously in infants. We also considered the percentage of coincidental contralateral spindles less arbitrary than the 2-second rule for the synchrony calculation. Along with asymmetry, from infancy to approximately 2 years of age, spindle duration was reported to reach its maximum between 3 and 5 months of age, decreasing thereafter [8, 45, 81]. In keeping with these findings, spindle duration in our group showed a weak but statistically significant association to PMA with a decrease in the short period of time from 55 + 1 to 63 weeks PMA (Table 4), for each week of PMA, there is a decrease of 0.09 s in the spindle duration (*p* = .018). However, after controlling for sex and timing of recording, (results not included) correlations lost statistical significance.

We also found that interhemispheric synchrony of sleep spindles was weakly correlated with GA (Table 4) with synchrony decreasing 1.31% for each increment of a week on the GA at birth (Table 4). However, this correlation was lost after correcting for PNA, sex, and time of recording.

### Relationship between sex and sleep parameters

From simple linear regression analysis, spectral power was 25.4% lower (*p* = .022) and BSI 23.9% (*p* = .010) higher in males. These values changed to 24.5% (*p* = .032) lower and 24.2% (*p* = .011) higher in males respectively after controlling for GA, PNA, sex, and timing of the nap (Table 4). Gender differences in some sleep spindle parameters have previously been directly [28] and indirectly observed, both before [10] and after puberty [83] in older cohorts. Fast spindle density is higher in female cohorts [28] and may be partly related to menstrual cycle [84]. Also, the evolution of sleep spindle frequency and density across aging is different for both sexes [25]. To our knowledge, this is the first time that sex differences have been reported in sleep spindle power and spindle symmetry in infancy. These differences at this young age may be driven by different hormonal [85], and brain connectivity profiles [86]. In light of these findings, future studies should consider sex differences when studying sleep spindle power and symmetry properties in infants. No gender differences were found in the sleep macrostructure parameters.

### Analysis of the relationship between sleep pressure and sleep time, and sleep features

We compared the sleep macrostructure and sleep spindle parameters between the morning and the afternoon recordings to determine if the time of recordings and circadian rhythm influence sleep patterns during daytime. Results from univariable analysis indicated that spindle duration was 0.28 min shorter in the afternoon (*p* = .034) but, after correcting for sex, GA and PNA, the correlation was no longer significant (Table 4). Also, no differences were found in sleep macrostructure (Table 5). As expected, the proportions of REM/NREM sleep in our diurnal study were smaller than those reported during night sleep, although the duration of a typical sleep cycle is similar [54]. This nocturnal/diurnal contrast in REM and NREM sleep proportions contrast has been described previously not only in adults but also in infants by Louis *et al.* [59] and might be at least partially related to the oscillation of body temperature across the day [87] (for a review [88]).

Similarly, although some macrostructural sleep elements are expected to change across sleep [88], no changes from the first to second sleep cycle were observed in this study which might be due to the narrow sleep time-frame of our analysis (Table 6). Adult studies have shown sleep spindles to be sensitive to reductions in sleep pressure through consecutive sleep cycles [23, 25, 28] and increases following sleep deprivation [24, 27, 89]. In our infants (Table 6), one sleep spindle parameter, spectral power, decreased in response to a decrease in sleep pressure from the first to the second NREM period. This contrasts with adult studies that found an increase in the spindle-related spectral power concurrent with a decrease in delta activity across sleep cycles [27, 90]. No studies to date have investigated spindle spectral power across consecutive sleep cycles in infants and Louis *et al*. showed no significant changes in amplitude across cycles in 12 infants studied [8]; hence this is an area that warrants further research.

### Strengths

We used polysomnography in this study, which is considered the gold-standard for sleep studies; additionally, good spatial coverage was assured by the use of 21 active electrodes. Recording in the sleep laboratory allowed for a standardized environment and control for factors such as light, noise, and temperature. A clinical physiologist was present at all times during the recording to address any technical problems.

All infants had a Griffiths-III assessment performed by certified clinical neuropsychologist/pediatricians at 18-months, ensuring that the infants included in this study had no neurodevelopmental impairment.

Spindles were identified as discrete features and selected for analysis manually by an experienced clinical physiologist which would be considered the gold standard for spindle detection and allowed correct detection of the spindle segments, as opposed to spectral power analysis of sigma frequencies or more rudimentary spindle-detection algorithms. High agreement scores were obtained when comparing a subset of these EEGs with the markings from an EEG-trained research nurse.

The present study included a large cohort of infants and described the sleep macrostructure at 4 to 5 months of age and is the biggest infant cohort reported to date describing sleep spindles and this will allow comparison with futures studies on maturation and neurodevelopment.

Our study resulted in the detailed description of the sleep spindles using parameters that generally are not studied together: frequency, duration, spectral power, brain symmetry index, synchrony, number and density, permitting a broader picture of the several elements that constitute the sleep spindles. To our knowledge, this was also the first study to report sex differences in sleep spindle parameters in infancy and future studies relating sleep spindles to neurodevelopmental pathologies will have to take account of those differences.

Spindles result from complex interactions between the thalamus and the cortex; they depend on the numerical extent of cortical neurons involved as well as the cortical layers involved and pathways [91]. Surface EEG, in turn, is only able to detect post-synaptic potentials from the most superficial region of the cortex. Detection of potentials is also dependent on the position of the neurons, as biological dipoles, on the cortical gyri, and the number of synchronized firing neurons [92]. As a result, only a fraction of the spindle oscillations generated are indirectly detectable by this technique. Nevertheless, the study of spindles with EEG is an accessible, real-time, noninvasive technique with promising results reflecting cognitive abilities [10, 16, 20, 21, 83] and neurologic conditions [36–41]

### Limitations

There are a number of limitations to our study. First, we could not control for the fact that some of the infants fell asleep during the trip to the sleep lab; effects of a short nap during the car journey may have affected sleep pressure. As seen in Table 6, sleep spindle power changes from the first to the second cycle. For this reason, a possible reduction of sleep pressure resulting from a nap during the trip to our lab may have affected sleep spindle power.

Although sleep spindle identification kappa scores were high between two raters, sleep staging was only performed by one neurophysiology trained rater and this may constitute a limitation to our study.

Despite being the biggest cohort of infants reported to date which provides a detailed analysis of sleep spindles, the number of infants may still be a limitation for group comparisons by gender and time of the day and particularly for comparisons between the first and second sleep cycle. Given the sample size, only medium/large effect sizes could be detected.

It was only possible to record one polysomnography per infant. Due to this, there was no previous infant adaptation to the laboratory. Results from first recording sessions may differ from results from other consecutive sessions [60]. Regarding sleep spindles, it is accepted that, based on the EEG spectra, they are fairly constant and represent an individual signature [15]. In a study of twelve adolescents, not all parameters presented good intra-individual reliability with one single recording; those parameters were slow spindle density and slow spindle duration [93]. There is no study so far on the intra-individual reliability of sleep spindles in infants. However, considering that only slow sleep spindles showed lower intra-individual reliability in adolescents [93] and that these types of spindles tend to appear toward toddlerhood [12], we do not believe this had a major impact on our sleep spindle analysis.

To conclude, in this study we characterize normative values for daytime sleep macrostructure and sleep spindles from 4 to 5 months of age. Spindle spectral power decreased from the first to the second sleep cycle. Sex differences in the sleep spindle spectral power and symmetry were the only independent variables studied from the first sleep cycle that remained statistically significant after controlling for GA and PNA at the recording, and time of nap. For this reason, sex differences should be taken into consideration in future studies in this age group. These values may provide a benchmark for future studies focusing on early recognition of risk of atypical neurodevelopmental trajectory.

## References

[1] Berry RB , et al The AASM Manual for the Scoring of Sleep and Associated Events: Rules, Terminology and Technical Specifications. Version 2.4. Darien, IL: American Academy of Sleep and Associated Events; 2017.

[2] Steriade M , et al The deafferented reticular thalamic nucleus generates spindle rhythmicity. J Neurophysiol. 1987;57(1):260–273. 3559675 10.1152/jn.1987.57.1.260

[3] Destexhe A , et al Modeling the control of reticular thalamic oscillations by neuromodulators. Neuroreport. 1994;5(17):2217–2220. 7881030 10.1097/00001756-199411000-00003

[4] Fuentealba P , et al The reticular nucleus revisited: intrinsic and network properties of a thalamic pacemaker. Prog Neurobiol. 2005;75(2):125–141. 15784303 10.1016/j.pneurobio.2005.01.002

[5] McCormick DA , et al Sleep and arousal: thalamocortical mechanisms. Annu Rev Neurosci. 1997;20:185–215. 9056712 10.1146/annurev.neuro.20.1.185

[6] Bonjean M , et al Corticothalamic feedback controls sleep spindle duration in vivo. J Neurosci. 2011;31(25):9124–9134. 21697364 10.1523/JNEUROSCI.0077-11.2011 PMC3131502

[7] Contreras D , et al Control of spatiotemporal coherence of a thalamic oscillation by corticothalamic feedback. Science. 1996;274(5288):771–774. 8864114 10.1126/science.274.5288.771

[8] Louis J , et al Ontogenesis of nocturnal organization of sleep spindles: a longitudinal study during the first 6 months of life. Electroencephalogr Clin Neurophysiol. 1992;83(5):289–296. 1385085 10.1016/0013-4694(92)90088-y

[9] Gibbs FA , et al Atlas of Electroencephalography. Vol. 1. Cambridge, MA: Addison-Wesley; 1950.

[10] Ujma PP , et al Sleep spindles and intelligence in early childhood-developmental and trait-dependent aspects. Dev Psychol. 2016;52(12):2118–2129. 27893249 10.1037/dev0000233

[11] Shinomiya S , et al Development of sleep spindles in young children and adolescents. Clin Electroencephalogr. 1999;30(2):39–43. 10358781 10.1177/155005949903000203

[12] D’Atri A , et al Different maturational changes of fast and slow sleep spindles in the first four years of life. Sleep Med. 2018;42:73–82. 29458750 10.1016/j.sleep.2017.11.1138

[13] Andrillon T , et al Sleep spindles in humans: insights from intracranial EEG and unit recordings. J Neurosci. 2011;31(49):17821–17834. 22159098 10.1523/JNEUROSCI.2604-11.2011 PMC3270580

[14] Zeitlhofer J , et al Topographic distribution of sleep spindles in young healthy subjects. J Sleep Res. 1997;6(3):149–155. 9358392 10.1046/j.1365-2869.1997.00046.x

[15] De Gennaro L , et al An electroencephalographic fingerprint of human sleep. Neuroimage. 2005;26(1):114–122. 15862211 10.1016/j.neuroimage.2005.01.020

[16] Bódizs R , et al Prediction of general mental ability based on neural oscillation measures of sleep. J Sleep Res. 2005;14(3):285–292. 16120103 10.1111/j.1365-2869.2005.00472.x

[17] Zygierewicz J , et al High resolution study of sleep spindles. Clin Neurophysiol. 1999;110(12):2136–2147. 10616119 10.1016/s1388-2457(99)00175-3

[18] Mölle M , et al Fast and slow spindles during the sleep slow oscillation: disparate coalescence and engagement in memory processing. Sleep. 2011;34(10):1411–1421. doi:10.5665/SLEEP.1290. 21966073 PMC3174843

[19] Schabus M , et al Hemodynamic cerebral correlates of sleep spindles during human non-rapid eye movement sleep. Proc Natl Acad Sci USA. 2007;104(32):13164–13169. 17670944 10.1073/pnas.0703084104 PMC1941810

[20] Hoedlmoser K , et al Slow sleep spindle activity, declarative memory, and general cognitive abilities in children. Sleep. 2014;37(9):1501–1512. doi:10.5665/sleep.4000. 25142558 PMC4153050

[21] Schabus M , et al Sleep spindle-related activity in the human EEG and its relation to general cognitive and learning abilities. Eur J Neurosci. 2006;23(7):1738–1746. 16623830 10.1111/j.1460-9568.2006.04694.x

[22] Cox R , et al Local sleep spindle modulations in relation to specific memory cues. Neuroimage. 2014;99:103–110. 24852461 10.1016/j.neuroimage.2014.05.028

[23] Bódizs R , et al The individual adjustment method of sleep spindle analysis: methodological improvements and roots in the fingerprint paradigm. J Neurosci Methods. 2009;178(1):205–213. 19061915 10.1016/j.jneumeth.2008.11.006

[24] De Gennaro L , et al The electroencephalographic fingerprint of sleep is genetically determined: a twin study. Ann Neurol. 2008;64(4):455–460. 18688819 10.1002/ana.21434

[25] Martin N , et al Topography of age-related changes in sleep spindles. Neurobiol Aging. 2013;34(2):468–476. 22809452 10.1016/j.neurobiolaging.2012.05.020

[26] Himanen SL , et al Spindle frequencies in sleep EEG show U-shape within first four NREM sleep episodes. J Sleep Res. 2002;11(1):35–42. 11869425 10.1046/j.1365-2869.2002.00273.x

[27] Dijk DJ , et al Dynamics of electroencephalographic sleep spindles and slow wave activity in men: effect of sleep deprivation. Brain Res. 1993;626(1–2):190–199. 8281430 10.1016/0006-8993(93)90579-c

[28] Purcell SM , et al Characterizing sleep spindles in 11,630 individuals from the National Sleep Research Resource. Nat Commun. 2017;8:15930. 28649997 10.1038/ncomms15930 PMC5490197

[29] Ujma PP , et al Nap sleep spindle correlates of intelligence. Sci Rep. 2015;5:17159. 26607963 10.1038/srep17159 PMC4660428

[30] Morin A , et al Motor sequence learning increases sleep spindles and fast frequencies in post-training sleep. Sleep. 2008;31(8):1149–1156. doi:10.5665/sleep/31.8.1149. 18714787 PMC2542961

[31] Clemens Z , et al Twenty-four hours retention of visuospatial memory correlates with the number of parietal sleep spindles. Neurosci Lett. 2006;403(1-2):52–56. 16714084 10.1016/j.neulet.2006.04.035

[32] Meier-Koll A , et al Walking through a maze alters the architecture of sleep. Percept Mot Skills. 1999;88(3 Pt 2):1141–1159. 10485095 10.2466/pms.1999.88.3c.1141

[33] Shimizu RE , et al Closed-loop targeted memory reactivation during sleep improves spatial navigation. Front Hum Neurosci. 2018;12:28. 29467633 10.3389/fnhum.2018.00028 PMC5808124

[34] Clemens Z , et al Overnight verbal memory retention correlates with the number of sleep spindles. Neuroscience. 2005;132(2):529–535. 15802203 10.1016/j.neuroscience.2005.01.011

[35] Hahn M , et al Developmental changes of sleep spindles and their impact on sleep-dependent memory consolidation and general cognitive abilities: A longitudinal approach. Dev Sci. 2019;22(1):e12706. 30252185 10.1111/desc.12706 PMC6492121

[36] Rauchs G , et al Is there a link between sleep changes and memory in Alzheimer’s disease? Neuroreport. 2008;19(11):1159–1162. 18596620 10.1097/WNR.0b013e32830867c4 PMC2925139

[37] Christensen JA , et al Sleep spindle alterations in patients with Parkinson’s disease. Front Hum Neurosci. 2015;9:233. 25983685 10.3389/fnhum.2015.00233 PMC4416460

[38] Merikanto I , et al ADHD symptoms are associated with decreased activity of fast sleep spindles and poorer procedural overnight learning during adolescence. Neurobiol Learn Mem. 2019;157:106–113. 30553020 10.1016/j.nlm.2018.12.004

[39] Gorgoni M , et al Sleep EEG oscillations in neurodevelopmental disorders without intellectual disabilities. Sleep Med Rev. 2020;49:101224. 31731102 10.1016/j.smrv.2019.101224

[40] Schilling C , et al Fast sleep spindle reduction in schizophrenia and healthy first-degree relatives: association with impaired cognitive function and potential intermediate phenotype. Eur Arch Psychiatry Clin Neurosci. 2017;267(3):213–224. 27565806 10.1007/s00406-016-0725-2

[41] Ferrarelli F , et al Reduced sleep spindle activity in schizophrenia patients. Am J Psychiatry. 2007;164(3):483–492. 17329474 10.1176/ajp.2007.164.3.483

[42] Shibagaki M , et al Nocturnal sleep in mentally retarded infants with cerebral palsy. Electroencephalogr Clin Neurophysiol. 1985;61(6):465–471. 2415320 10.1016/0013-4694(85)90964-2

[43] Krol A , et al Thalamic reticular dysfunction as a circuit endophenotype in neurodevelopmental disorders. Neuron. 2018;98(2):282–295. 29673480 10.1016/j.neuron.2018.03.021 PMC6886707

[44] Novelli L , et al Mapping changes in cortical activity during sleep in the first 4 years of life. J Sleep Res. 2016;25(4):381–389. 26854271 10.1111/jsr.12390

[45] Scholle S , et al Sleep spindle evolution from infancy to adolescence. Clin Neurophysiol. 2007;118(7):1525–1531. 17475551 10.1016/j.clinph.2007.03.007

[46] Rosanova M , et al Pattern-specific associative long-term potentiation induced by a sleep spindle-related spike train. J Neurosci. 2005;25(41):9398–9405. 16221848 10.1523/JNEUROSCI.2149-05.2005 PMC6725710

[47] Clawson BC , et al Form and function of sleep spindles across the lifespan. Neural Plast. 2016;2016:6936381. 27190654 10.1155/2016/6936381 PMC4848449

[48] Gruber R , et al The association between sleep spindles and IQ in healthy school-age children. Int J Psychophysiol. 2013;89(2):229–240. 23566888 10.1016/j.ijpsycho.2013.03.018

[49] Chatburn A , et al Sleep spindle activity and cognitive performance in healthy children. Sleep. 2013;36(2):237–243. doi:10.5665/sleep.2380. 23372271 PMC3543056

[50] Geiger A , et al The sleep EEG as a marker of intellectual ability in school age children. Sleep. 2011;34(2):181–189. doi:10.1093/sleep/34.2.181. 21286251 PMC3022938

[51] Page J , et al Social, motor, and cognitive development through the lens of sleep network dynamics in infants and toddlers between 12 and 30 months of age. Sleep. 2018;41(4). doi:10.1093/sleep/zsy024 PMC6018907 29506060

[52] Fogel SM , et al Sleep spindles and learning potential. Behav Neurosci. 2007;121(1):1–10. 17324046 10.1037/0735-7044.121.1.1

[53] Fogel S , et al Sleep spindles: a physiological marker of age-related changes in gray matter in brain regions supporting motor skill memory consolidation. Neurobiol Aging. 2017;49:154–164. 27815989 10.1016/j.neurobiolaging.2016.10.009

[54] Ficca G , et al Sleep organization in the first year of life: developmental trends in the quiet sleep-paradoxical sleep cycle. J Sleep Res. 2000;9(1):1–4. 10.1046/j.1365-2869.2000.00172.x 10733682

[55] Dan B , et al A neurophysiological perspective on sleep and its maturation. Dev Med Child Neurol. 2006;48(9):773–779. 16904027 10.1017/S0012162206001654

[56] Dereymaeker A , et al Review of sleep-EEG in preterm and term neonates. Early Hum Dev. 2017;113:87–103. 28711233 10.1016/j.earlhumdev.2017.07.003 PMC6342258

[57] Schulz H , et al REM latency: development in the first year of life. Electroencephalogr Clin Neurophysiol. 1983;56(4):316–322. 6193945 10.1016/0013-4694(83)90257-2

[58] Luca G , et al Age and gender variations of sleep in subjects without sleep disorders. Ann Med. 2015;47(6):482–491. 26224201 10.3109/07853890.2015.1074271

[59] Louis J , et al Sleep ontogenesis revisited: a longitudinal 24-hour home polygraphic study on 15 normal infants during the first two years of life. Sleep. 1997;20(5):323–333. doi:10.1093/sleep/20.5.323. 9381053

[60] Grigg-Damberger M , et al The visual scoring of sleep and arousal in infants and children. J Clin Sleep Med. 2007;3(2):201–240. 17557427

[61] Vyazovskiy VV , et al Sleep and the single neuron: the role of global slow oscillations in individual cell rest. Nat Rev Neurosci. 2013;14(6):443–451. 23635871 10.1038/nrn3494 PMC3972489

[62] Bertrand SJ , et al Transient neonatal sleep fragmentation results in long-term neuroinflammation and cognitive impairment in a rabbit model. Exp Neurol. 2020;327:113212. 31987835 10.1016/j.expneurol.2020.113212

[63] Tononi G , et al Sleep and the price of plasticity: from synaptic and cellular homeostasis to memory consolidation and integration. Neuron. 2014;81(1):12–34. 24411729 10.1016/j.neuron.2013.12.025 PMC3921176

[64] Kurth S , et al Sleep and early cortical development. Curr Sleep Med Rep. 2015;1(1):64–73. 26807347 10.1007/s40675-014-0002-8 PMC4721216

[65] Morrissey MJ , et al Active sleep and its role in the prevention of apoptosis in the developing brain. Med Hypotheses. 2004;62(6):876–879. 15142640 10.1016/j.mehy.2004.01.014

[66] Li W , et al REM sleep selectively prunes and maintains new synapses in development and learning. Nat Neurosci. 2017;20(3):427–437. 28092659 10.1038/nn.4479 PMC5535798

[67] Khazipov R , et al Early motor activity drives spindle bursts in the developing somatosensory cortex. Nature. 2004;432(7018):758–761. 15592414 10.1038/nature03132

[68] Blumberg MS , et al Spatiotemporal structure of REM sleep twitching reveals developmental origins of motor synergies. Curr Biol. 2013;23(21):2100–2109. 24139739 10.1016/j.cub.2013.08.055 PMC3823644

[69] Horvath K , et al Memory in 3-month-old infants benefits from a short nap. Dev Sci. 2018;21(3):e12587. 28722249 10.1111/desc.12587

[70] Friedrich M , et al The sleeping infant brain anticipates development. Curr Biol. 2017;27(15):2374–2380.e3. 28756948 10.1016/j.cub.2017.06.070

[71] Simon KNS , et al Sleep confers a benefit for retention of statistical language learning in 6.5month old infants. Brain Lang. 2017;167:3–12. 27291337 10.1016/j.bandl.2016.05.002

[72] Mindell JA , et al Sleep, mood, and development in infants. Infant Behav Dev. 2015;41:102–107. 26386882 10.1016/j.infbeh.2015.08.004

[73] Sadeh A , et al Sleep in infancy and childhood: implications for emotional and behavioral difficulties in adolescence and beyond. Curr Opin Psychiatry. 2014;27(6):453–459. 25247458 10.1097/YCO.0000000000000109

[74] van Putten MJ . The revised brain symmetry index. Clin Neurophysiol. 2007;118(11):2362–2367. 17888719 10.1016/j.clinph.2007.07.019

[75] O’Toole JM , et al NEURAL: quantitative features for newborn EEG using Matlab. 2017;arXiv:1704.05694v05691.

[76] Stroud L , et al Griffiths III Manual. Part 1: Overview, Development and Psychometric Properties. 3rd ed. Oxford, UK: Hogrefe Ltd; 2016.

[77] de Weerd AW , et al The development of sleep during the first months of life. Sleep Med Rev. 2003;7(2):179–191. 12628217 10.1053/smrv.2002.0198

[78] Paavonen EJ , et al Normal sleep development in infants: findings from two large birth cohorts. Sleep Med. 2020;69:145–154. 32087408 10.1016/j.sleep.2020.01.009

[79] Oliviero B , et al Longitudinal study of sleep behavior in normal infants during the first year of life. J Clin Sleep Med. 2014;10(10):1119–1127. 25317093 10.5664/jcsm.4114 PMC4173090

[80] Cox R , et al Individual differences in frequency and topography of slow and fast sleep spindles. Front Hum Neurosci. 2017;11:433. 28928647 10.3389/fnhum.2017.00433 PMC5591792

[81] Hughes JR . Development of sleep spindles in the first year of life. Clin Electroencephalogr. 1996;27(3):107–115. 8828973 10.1177/155005949602700303

[82] Yordanova J , et al. Sleep spindles in the right hemisphere support awareness of regularities and reflect pre-sleep activations. Sleep. 2017;40(11). doi:10.1093/sleep/zsx151. PMC5806558 28958008

[83] Bódizs R , et al Sleep spindling and fluid intelligence across adolescent development: sex matters. Front Hum Neurosci. 2014;8:952. 25506322 10.3389/fnhum.2014.00952 PMC4246682

[84] Driver HS , et al Sleep and the sleep electroencephalogram across the menstrual cycle in young healthy women. J Clin Endocrinol Metab. 1996;81(2):728–735. 8636295 10.1210/jcem.81.2.8636295

[85] Kelava I , et al Male sex hormones increase excitatory neuron production in developing human neocortex. bioRxiv. 2020; 2020.2010.2024.353359.

[86] Yap PT , et al Development trends of white matter connectivity in the first years of life. PLoS One. 2011;6(9):e24678. 21966364 10.1371/journal.pone.0024678 PMC3179462

[87] Charles AC , et al Timing of REM sleep is coupled to the circadian rhythm of body temperature in man. Sleep. 1980;2(3):329–346. doi:10.1093/sleep/2.3.329. 7403736

[88] Le Bon O . Relationships between REM and NREM in the NREM-REM sleep cycle: a review on competing concepts. Sleep Med. 2020;70:6–16. 32179430 10.1016/j.sleep.2020.02.004

[89] De Gennaro L , et al Effect of slow-wave sleep deprivation on topographical distribution of spindles. Behav Brain Res. 2000;116(1):55–59. 11090885 10.1016/s0166-4328(00)00247-3

[90] Werth E , et al Spindle frequency activity in the sleep EEG: individual differences and topographic distribution. Electroencephalogr Clin Neurophysiol. 1997;103(5):535–542. 9402884 10.1016/s0013-4694(97)00070-9

[91] Krishnan GP , et al Thalamocortical and intracortical laminar connectivity determines sleep spindle properties. PLoS Comput Biol. 2018;14(6):e1006171. 29949575 10.1371/journal.pcbi.1006171 PMC6039052

[92] Cooper R , et al Techniques in Clinical Neurophysiology: A Practical Manual. London: Elsevier Churchill Livingstone; 2005:28–30.

[93] Reynolds CM , et al Reliability of sleep spindle measurements in adolescents: how many nights are necessary? J Sleep Res. 2019;28(1):e12698. 29736916 10.1111/jsr.12698

[94] Engle WA . Age terminology during the perinatal period. Pediatrics. 2004;114(5):1362– 1364. 15520122 10.1542/peds.2004-1915

